# The Generalization of Conscious Attentional Avoidance in Response to Threat Among Breast Cancer Women With Persistent Distress

**DOI:** 10.3389/fpsyg.2020.589088

**Published:** 2020-12-21

**Authors:** Danielle Wing Lam Ng, Richard Fielding, Wendy Wing Tak Lam

**Affiliations:** ^1^Division of Behavioural Sciences, Centre for Psycho-Oncological Research and Training, School of Public Health, The University of Hong Kong, Hong Kong, China; ^2^Li Ka Shing Faculty of Medicine, Jockey Club Institute of Cancer Care, The University of Hong Kong, Hong Kong, China

**Keywords:** psycho-oncology, attentional bias, interpretation bias, psychological distress, breast cancer

## Abstract

**Objectives:**

A sample of women with persistent distress following breast cancer (BC) previously exhibited attentional bias (AB) away from supraliminally presented cancer-or threat-related information, responses consistent with avoidance coping, and showed negative interpretation bias. Here, we attempt to characterize the nature of supraliminal AB and interpretation bias in that sample of women by comparing against healthy controls.

**Methods:**

Extending our previous work, we compared AB patterns for supraliminally presented negatively valenced words and cancer-related information (CRI) assessed by modified dot-probe tasks and negative interpretation bias assessed by an ambiguous cue task between 140 BC women previously identified as featuring low-stable or persistent high anxiety and 150 age-matched non-BC healthy controls having HADS-defined low or high anxiety (HADS-anxiety scores = 8).

**Results:**

Attentional avoidance of non-cancer-related negatively valenced words was seen among the anxious BC group, while heightened attention toward such words was seen in anxious healthy controls, *F*(3, 282) = 3.97, *p* = 0.009. However, all anxious women in both BC and healthy groups directed attention away from CRI under supraliminal conditions. Interpretation bias scores were significantly higher in BC women with high anxiety vs. healthy controls with high anxiety, *F*(3, 282) = 13.26, *p* < 0.001.

**Conclusion:**

Women with high anxiety generalized conscious attentional avoidance responses to negatively valenced stimuli, indicating a likely hypersensitivity to potential threat in ambiguous cues and exaggerated threat perception. This may cause (or reflect) maladaptive emotional regulation. Attention focus training, reducing threat salience or modifying threat appraisal, may help women alleviate anxiety levels after BC.

## Introduction

Though many women with breast cancer (BC) experience stable low or transient distress, a subset, ∼10–20%, remain distressed > 5 years after diagnosis, detrimentally impacting their quality of life (Helgeson et al., 2004; Burgess et al., 2005; Henselmans et al., 2010; Lam et al., 2010, 2012). Understanding individual differences in psychological adjustment to BC helps enable accurate identification of those at high risk of persistent distress and informs better therapeutic approaches (Lam et al., 2018).

Cognitive theories of emotional disorders claim that biased attentional and interpretation processing of threat-relevant information underpins enhanced vulnerability toward developing symptoms of anxiety and/or depression by modifying information salience, which may involve an interplay between early, automatic (preconscious) and later, strategic (conscious) stages of processing (Mathews and MacLeod, 2005; Bar-Haim et al., 2007). Prior findings in emotional disorders using experimental paradigms including dot-probe tasks better capture the time-course effect of attentional bias (AB) by manipulating stimuli exposure duration. Such studies reported that individuals with anxiety often displayed facilitated preconscious (automatic) attentional allocation (bias) toward threat-related stimuli under initial subliminal exposure, which was either maintained or reallocated away from such stimuli, depending on the emotional condition, under prolonged supraliminal exposures reflecting conscious control; in depression, AB toward threat-related stimuli appeared to be confined to only supraliminal exposures, indicative of inability to disengage among depressed individuals (Mathews and MacLeod, 2005; Bar-Haim et al., 2007; Cisler and Koster, 2010).

Few studies have adopted dot-probe tasks to identify the distinct temporal nature of AB in psychological distress among cancer survivors. In the only study found on subliminal AB, subliminal bias was uncorrelated with distress among BC survivors (Glinder et al., 2007). The findings from four available studies on supraliminal AB were inconclusive, with one reporting AB away from cancer-related word stimuli (Glinder et al., 2007) among those with higher distress, one reporting no significant association of supraliminal AB with fear of cancer recurrence (Butow et al., 2015), and the others reporting that cancer patients exhibiting significant supraliminal AB toward negatively valenced facial expression experienced higher anxiety (Koizumi et al., 2018) or greater symptoms of posttraumatic stress disorder (PTSD) (Chan et al., 2013). However, due to the use of cross-sectional study design, none have examined AB in distress with reliable distress chronicity assessment.

Our previous AB study (Lam et al., 2018) sampled eligible Chinese women with BC from an ongoing longitudinal study of psychological distress trajectories following BC diagnosis. We found that women with persistent distress exhibited a bias away from supraliminally presented (1,250 ms) negatively valenced or cancer-related stimuli (avoidance) in a neutral prime condition; women with low-stable/transient distress showed a bias toward the stimuli (vigilance) (Lam et al., 2018). Such supraliminal attentional response shared the distinctive patterns of attentional avoidant expression reported in a PTSD sample (Bar-Haim et al., 2010). Intentional threat-information avoidance could impede adaptive engagement coping to manage threat, thereby enhancing psychological distress (Glinder et al., 2007; Lam et al., 2018). However, the absence of a non-BC control rendered the specificity of our findings in BC patients unclear and so warrants further investigation.

To test the specificity of these results to cancer threat, we compared patterns of AB expression in response to supraliminally exposed negatively valenced stimuli among women recovering from BC who reported persistently high anxiety and healthy women reporting high anxiety. Considering cognitive theories of emotional disorders and the findings from our previous study, we hypothesized that both groups of anxious women would exhibit conscious attentional avoidance of supraliminally presented negatively valenced stimuli (hypothesis 1). However, the personal relevance hypothesis posits that AB may be thematically specific (MacLeod and Rutherford, 1992; Pergamin-Hight et al., 2015). We therefore tested for differences in the patterns of AB for supraliminally presented cancer-related information (CRI) between both groups of anxious women, hypothesizing that the BC group would display greater consciously averted AB under this condition (hypothesis 2). In addition, schema theories of emotional disorders propose that the priming effect of AB acts via schema-driven processing (Beck, 1979; Beck and Clark, 1997). Assuming a more elaborated cancer-specific schemas in the BC sample due to their cancer experience that could be accessed and activated easily reinforcing CRI processing, we hypothesized that the BC group would display greater consciously averted AB under cancer-related prime compared with the non-BC control (hypothesis 3).

Negative interpretation bias for ambiguous information is also common in emotional disorders (Mathews and MacLeod, 2005), individuals with chronic pain (Edwards and Pearce, 1994; Pincus et al., 1996) and with chronic fatigue syndrome (Moss-Morris and Petrie, 2003). Similarly, a significant positive correlation between negative interpretation bias and anxiety was observed in our earlier study; following BC, women with persistent anxiety tended to disambiguate information in a negative, cancer-related manner, relative to their low-stable anxiety peers (Lam et al., 2018). To clarify interpretation bias in anxious BC survivors, we compared differences in negative interpretation bias between BC and healthy women with high anxiety, hypothesizing that following BC, women with high anxiety relative to their healthy counterparts would be predisposed to cancer-related interpretations of ambiguous information (hypothesis 4).

## Materials and Methods

### Participants and Design

Ethical approval of this study was obtained from participating institutions (Ref: UW14-136).

A total of 140 Cantonese-speaking Chinese women previously diagnosed with and treated for non-metastatic BC {67 with persistent distress and 73 with low-stable distress [based on the Mixture Growth Modeling (MGM) classification using the 14-item Hospital Anxiety and Depression Scale (HADS)] (Snaith and Zigmond, 1986; Leung et al., 1999)} were recruited for the original AB study (Lam et al., 2018) from an earlier ongoing longitudinal study. Of the 67 subjects demonstrating persistent distress in the ongoing longitudinal study, only 31 (46.3%) continued to report high HADS-anxiety scores (HADS-anxiety scores ≥ 8) on recruitment into the original AB study (Lam et al., 2018). These women also formed the BC sample for the present study. Details of the BC sample recruitment and the MGM characterization of distress trajectories are described in Lam et al. (2018).

To clarify whether the observed conscious attentional avoidance of supraliminally presented threat-related information in the original AB study defined a pattern of AB specific to persistent high anxious BC survivors (Lam et al., 2018), the current study used the data previously presented in Lam et al. (2018) and additionally included a comparison group of 150 age-matched healthy women without history of cancer or mental health problems recruited from the University of Hong Kong via bulk emailed advertisement. Potential participants were contacted by phone with a detailed explanation of the study. Using a HADS-anxiety cutoff score of >8 to classify high vs. low anxiety levels (Snaith and Zigmond, 1986; Leung et al., 1999), 17 (11.3%) healthy controls reported high anxiety, whereas 133 (88.7%) reported low anxiety.

Subsequent analyses compared four anxiety groups: breast cancer sample—anxious BC (*N* = 31) or non-anxious BC (*N* = 109), and healthy controls—anxious HC (*N* = 17) or non-anxious HC (*N* = 133).

### Measures

#### Psychological Distress

The Chinese version of HADS (Snaith and Zigmond, 1986; Leung et al., 1999), including two 7-item subscales, was employed to assess anxiety and depression, respectively. Each item is rated on a four-point Likert-type scale, with higher scores indicating greater psychological distress. The HADS cutoff score of 8 was used to indicate high anxiety. Good internal consistency of each subscale was reported (anxiety, Cronbach’s *α* = 0.89; depression, Cronbach’s *α* = 0.85).

#### Attentional Bias Paradigm

Attentional bias for negatively valenced stimuli and CRI was measured by two modified dot-probe tasks (MacLeod et al., 1986) using visually presented word stimuli. The paradigm was presented on a 15.6 inch laptop using E-Prime, positioned approximately 50 cm away from the participants. The assessment was conducted in a quiet private room with minimum distraction. The dot-probe task demonstrated poor internal consistency with Cronbach’s *α* ranging between -0.42 and 0.26, suggesting high variability in response to the individual trials of the task.

#### Negatively Valenced Stimuli

A word list comprised of 64 neutral (e.g., “Height”), 32 negative (e.g., “Suicide”), and 32 positive (e.g., “Happiness”) two-character Chinese compound words chosen from validated word-sets based on their valence and arousal ratings (Bleiker et al., 2000; Lee et al., 2008; Leung et al., 2009) was used in this dot-probe task (see Supplementary Materials).

Every trial began with a fixation for 500 ms, followed by a priming condition involving a subliminal presentation (20 ms) of a two-character Chinese compound word either “breast cancer” (target prime) or “sky” (neutral prime) (Bullock and Bonanno, 2013; Lam et al., 2018) to examine the content-specific nature of AB. Following a 500 ms pattern mask, a word pair, one negatively/positively valenced and one neutral, was presented horizontally for 1,250 ms (to assess later supraliminal, strategic attentional processing) (Mogg et al., 2004; Lam et al., 2018). A probe stimulus (dot) appeared in the location of one of the paired words immediately after the offset of each word pair. Response latency for probe detection was recorded.

Faster reaction times to probes replacing negatively valenced words (congruent events) vs. neutral words (incongruent events) indicate AB toward negatively valenced stimuli (Mogg et al., 2004; Lam et al., 2018). AB scores were calculated using the following formula (Butow et al., 2015):

$$B\,i\,a\,s\,i\,n\,d\,e\,x=\frac{\left[\left(t\,r\,p\,l-t\,l\,p\,l\right)+\left(t\,l\,p\,r-t\,r\,p\,r\right)\right]}{2}$$

where *t* = target word, *p* = probe location, *r* = right side, and *l* = left side. A positive bias score represents an AB toward the target stimuli, whereas a negative bias score represents an AB away from the target stimuli. Recorded reaction times from trials with errors and outliers of < 200 or > 3,000 ms, or more than 3 *SD* above each participants’ mean reaction time, were discarded from analysis (Bradley et al., 1997; Lam et al., 2018).

#### Cancer-Related Information

A similar modified dot-probe task with cancer-related words (e.g., “Cancer treatment”) replacing negative valenced words as target stimuli was used (Lam et al., 2018). In this second dot-probe task, 32 cancer-related two-character Chinese compound words that have been pilot-tested on BC patients and healthy women were used, and the priming condition was excluded since CRI was used as the target threat stimuli (see Supplementary Materials). In all other matters, the settings were the same as described above.

#### Ambiguous Cue Task

Interpretation bias for CRI was measured by an ambiguous cues task (Pincus et al., 1996; Moss-Morris and Petrie, 2003; Lam et al., 2018). The critical stimuli were 25 ambiguous and 25 unambiguous Chinese words (see Supplementary Materials). Each ambiguous word represents a pair of homophones, words that while sounding the same can have neutral or cancer-related meanings (e.g., 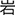 “rock” is phonetically similar to “cancer” in Chinese). All words were presented aurally in a random order. Participants were instructed to write down the first Chinese compound word they associated the cue with after the presentation of each word. Written responses were coded by two independent raters who were blinded to the participants’ anxiety status. One point was given for every cancer-related negative interpretation. Higher total scores suggest greater negative interpretation tendency for ambiguous cues. The two coders reached 100% agreement on scoring.

### Procedure

After obtaining fully informed consent, participating women completed the ambiguous cue task, two dot-probe tasks, and a set of questionnaires in that order.

### Data Analysis

To compare the patterns of supraliminal AB expression for threat-related information between BC and healthy control groups, a mixed repeated measures analysis of variance was conducted with the anxiety group as the between-subjects variable (anxious BC vs. non-anxious BC vs. anxious healthy controls vs. non-anxious healthy controls) and prime condition (target vs. neutral prime) and target word (positively vs. negatively valenced) as within-subjects variables. Univariate analysis of variance was conducted to compare group differences in interpretation bias scores.

## Results

### Sample Characteristics

Sociodemographics, except occupation (*χ*^2^ = 22.38, *p* < 0.001), did not significantly differentiate between women with BC and healthy controls. Women with BC were less likely to be retired or more likely to be unemployed than were healthy controls. Women with BC averaged 4.75 years since cancer diagnosis. Overall mean levels of anxiety (*p* = 0.004) and depression (*p* = 0.002) were higher in the BC group than in healthy controls (Table 1). Table 2 presents the demographic and psychological characteristics of BC and healthy control anxiety subgroups.

**TABLE 1:** Demographic and psychological characteristics of breast cancer survivors and healthy controls.

	Overall sample (*n* = 290)	Healthy controls (*n* = 150)	BC survivors (*n* = 140)	*χ* ^2^/*t*	*p*-value
**Demographic characteristics**					
Age (years), mean ± *SD*	55.58 ± 8.21	55.35 ± 8.32	55.81 ± 8.10	0.48	0.633
**Marital status, *n* (%)**				0.056	0.814
Single/divorced/separated/widowed	91 (31.4%)	48 (32.0%)	43 (30.7%)		
Married/cohabiting	199 (68.6%)	102 (68.0%)	97 (69.3%)		
**Education level**				2.43	0.119
No formal/primary tertiary	44 (15.2%)	18 (12.0%)	26 (18.6%)		
Secondary/tertiary	246 (84.8%)	132 (88.0%)	114 (81.4%)		
**Occupation status**				22.34	<0.001**
Employed (full-time/part-time)	157 (54.1%)	79 (52.7%)	78 (55.7%)		
Retired	58 (20.0%)	38 (25.3%)	20 (14.3%)		
Housewife	55 (19.0%)	31 (20.7%)	24 (17.1%)		
Unemployed	19 (6.6%)	1 (0.7%)	18 (12.9%)		
Others	1 (0.3%)	1 (0.7%)	0 (0%)		
**Clinical characteristics**					
Time since diagnosis (years) ± *SD*	–	–	4.75 ± 1.33		
Currently receiving any cancer treatments	–	–	83 (59.3%)		
Currently receiving hormonal therapy	–	–	79 (56.4%)		
Currently receiving target therapy	–	–	2 (1.4%)		
Currently receiving chemotherapy	–	–	2 (1.4%)		
Currently receiving radiotherapy	–	–	0 (0%)		
No active treatment	–	–	57 (40.7%)		
Cancer recurrence	–	–	6 (4.3%)		
Psychological distress					
HADS-anxiety, mean + *SD*		3.77 ± 3.50	5.14 ± 4.43	2.91	0.004*
HADS-depression, mean + *SD*		3.09 ± 3.20	4.43 ± 3.86	3.20	0.002*

BC, breast cancer; SD, standard deviation. *p < 0.05; **p < 0.001.

**TABLE 2:** Demographic and psychological characteristics of breast cancer and healthy control subgroups.

	Anxious BC (*n* = 31)	Anxious HC (*n* = 17)	Non-anxious BC (*n* = 109)	Non-anxious HC (*n* = 133)
**Demographic characteristics**				
Age (years), mean + *SD*	52.90 ± 7.29	56.18 ± 6.82	56.64 ± 8.16	55.25 ± 8.51
**Marital status, *n* (%)**				
Single/divorced/separated/widowed	11 (35.5%)	7 (41.2%)	32 (29.4%)	41 (30.8%)
Married/cohabiting	20 (64.5%)	10 (58.8%)	77 (70.6%)	92 (69.2%)
**Education level**				
No formal/primary tertiary	4 (12.9%)	3 (17.6%)	22 (20.2%)	15 (11.3%)
Secondary/tertiary	27 (87.1%)	14 (82.4%)	87 (79.8)	118 (88.7%)
**Occupation status**				
Employed (full-time/part-time)	16 (51.6%)	9 (52.9%)	62 (56.9%)	70 (52.6%)
Retired	0 (0%)	3 (17.6%)	20 (18.3%)	35 (26.3%)
Housewife	5 (16.1%)	5 (29.4%)	19 (17.4%)	26 (19.5%)
Unemployed	10 (32.3%)	0 (0%)	8 (7.3%)	1 (0.8%)
Others	0 (0%)	0 (0%)	0 (0%)	1 (0.8%)
**Psychological distress**				
HADS-anxiety, mean ± *SD*	11.48 ± 3.06	11.41 ± 2.83	3.34 ± 2.80	2.80 ± 2.09
HADS-depression, mean ± *SD*	9.03 ± 3.32	8.29 ± 2.87	3.12 ± 2.92	2.42 ± 2.57

**BC, breast cancer; HC, healthy controls; SD, standard deviation.**

### Attentional Bias for Negatively Valenced Stimuli

Since occupational status differed by group, this was included as a covariate in subsequent analyses.

**FIGURE 1:**
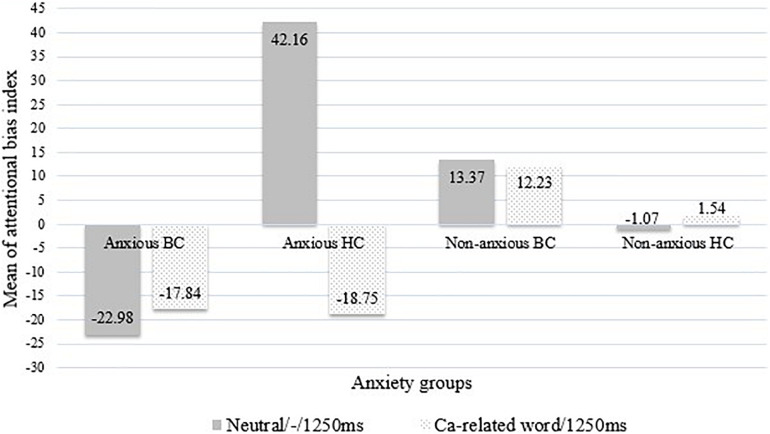
The patterns of supraliminal attentional bias for negatively valenced or cancer-related words among anxiety groups. BC, breast cancer; HC, healthy controls. Neutral/-/1,250 ms: using supraliminally presented negatively valenced words under neutral prime in the dot-probe task. Ca-related word/1,250 ms: using supraliminally presented cancer-related words in the dot-probe task. A positive bias score represents an attentional vigilance, whereas a negative bias score represents an attentional avoidance.

Repeated measures analysis of variance showed a significant main effect for groups, *F*(3, 282) = 3.06, *p* = 0.029, η^2^ = 0.032, and for target words, *F*(1, 282) = 4.38, *p* = 0.037, η^2^ = 0.015, and a significant three-way interaction of anxiety group × prime condition × target word, *F*(3, 282) = 2.74, *p* = 0.044, η^2^ = 0.028. *Post hoc* tests showed that there was no difference in AB in response to negatively valenced words vs. positively valenced words, and anxious BC subjects showed a significant bias away [mean = −22.98, *SD* 49.03, *F*(3, 282) = 3.97, *p* = 0.009] from supraliminally presented negatively valenced stimuli only under neutral prime, whereas anxious HC subjects had a bias toward such stimuli (mean = 42.16, *SD* 105.20; Figure 1).

### Attentional Bias for Cancer-Related Information

The observed marginal main effect for groups did not attain significance, *F*(3, 281) = 2.54, *p* = 0.056, but there was a significant main effect for target word, *F*(1, 281) = 5.39, *p* = 0.021, *η*^2^ = 0.019, and a significant two-way interaction between anxiety group × target word, *F*(3, 281) = 4.11, *p* = 0.007, *η*^2^ = 0.042. *Post hoc* tests indicated that, again, AB did not differ in response to cancer-related words vs. positively valenced words, and both anxious BC and anxious HC women showed AB away from supraliminally presented CRI (mean = −17.84, *SD* 52.81, for anxious BC vs. mean = −18.75, *SD* 29.89, for anxious HC, *p* > 0.05; Figure 1).

### Interpretation Bias

Univariate analysis of variance showed a significant main effect for cancer status, *F*(1, 282) = 29.69, *p* < 0.001, and a significant two-way interaction between cancer status and anxiety status, *F*(1, 282) = 4.24, *p* = 0.040. *Post hoc* testing showed a significant difference for interpretation bias scores in anxiety groups, *F*(3, 282) = 13.26, *p* < 0.001, on stimulus ambiguity. Anxious BC group members obtained significantly higher negative interpretation bias scores (mean = 7.19, *SD* 3.20, *p* < 0.001) than did anxious HC group members (mean = 4.22, *SD* 1.89). These scores were also higher for non-anxious BC group members (mean = 6.02, *SD* 2.38, *p* < 0.001) vs. non-anxious HC members (mean = 4.60, *SD* 2.16).

## Discussion

This study compared supraliminal AB expression among cancer patients and healthy controls classified by distress status, in response to cancer-specific and ambiguous threat-related information, providing several new insights. First, women with BC in general reported higher HADS-anxiety scores than did healthy controls, consistent with the meta-analysis study of Mitchell et al. (2013) suggesting that long-term cancer survivors are at increased risk of anxiety compared with healthy controls.

A comparable pattern of supraliminal AB for CRI was seen in both high anxiety groups, contradicting the hypothesized thematic specificity in AB (MacLeod and Rutherford, 1992; Custers et al., 2015; Pergamin-Hight et al., 2015). Women in both high anxiety groups exhibited conscious attentional avoidance of CRI, potentially reflecting a common self-protective coping response to stimuli with high threat value. Cancer, a life-threatening disease, is often encoded in cancer schemas as a “killer” on the basis of second-hand stories, observations, and even personal life experience. The activation of pre-existing cancer schemata by relevant information might automatically present high threat warnings especially in emotionally vulnerable individuals (Beck and Clark, 1997; Clark and Beck, 1999). Schema activation involves parallel cognition and emotional processing. Primary appraisal of cancer threat as lethal and minimally controllable likely evokes aversive emotions like fear. Under such circumstances, instrumental coping attempts may be seen as ineffective, and reliance on emotion-focused coping may ensue (Folkman, 2013). One strategy is to avoid stimuli that trigger the fear response by conscious reallocation of attention away from threatening stimuli. Motivated disengagement coping responses such as avoidance to downregulate unwanted emotions (Lazarus and Folkman, 1984; Stapinski et al., 2010) might interfere with threat habituation, thereby contributing to prolonged psychological distress (Glinder et al., 2007; Mogg and Bradley, 2016; Lam et al., 2018). Substantial evidence has demonstrated the association between disengagement coping and emotional dysfunction in both cancer (Lam et al., 2012; Brandão et al., 2017; Morris et al., 2018) and non-cancer populations (Aldao et al., 2010).

Another coping approach (or perhaps later stage) is to increase threat monitoring in an attempt to reduce uncertainty and hence threat imminence, manifesting as heightened health anxiety, which features enhanced distress. Interestingly, our findings identified a generalized pattern of supraliminal AB expression responding to general negatively valenced stimuli among women diagnosed with BC with high anxiety, in comparison with healthy controls with high anxiety. Women diagnosed with BC experiencing persistently high anxiety, again, exhibited conscious attentional avoidance of negatively valenced stimuli. Conversely, healthy controls with high anxiety showed conscious attentional vigilance toward the stimuli. Theoretically, exposure to a stressful life event (e.g., cancer diagnosis) might entrain (or reinforce pre-existing) dysfunctional schemas which embody enhanced anticipation of harm or perception of loss on next encounter, further reducing the activation threshold of threat-alert signaling in emotionally vulnerable cancer patients (Lazarus and Folkman, 1984; Beck and Clark, 1997; Clark and Beck, 1999). Threat stimuli may become overgeneralized and increasingly ambiguous. As such, affected women diagnosed with BC with high anxiety relative to anxious healthy controls might become disproportionately sensitive to negatively valenced stimuli that shared only vague similarities with CRI and overestimate threat or danger arising therefrom, prompting a generalization of maladaptive conscious attentional avoidance in response to general threatening information. Comparable but non-significant patterns were also seen in both groups with high depression (a considerable overlap exists between the anxious and depressed groups). However, the observed patterns may represent comorbid anxiety (Mathews and MacLeod, 2005), which was not examined here.

Despite no observed significant difference in the mean AB score reflecting patterns of supraliminal AB for CRI or negatively valenced stimuli between both non-anxious groups (Table 3), non-anxious healthy controls had near-zero scores, indicative of no significant bias (Mogg et al., 2004), whereas non-anxious women in the BC group showed relatively higher positive scores representing a tendency to direct attention toward threatening information. Modified attentional focus in non-anxious women with BC (but not in non-anxious healthy controls) provided indirect support for the notion that cancer experience may lower sensitivity threshold toward potential threat. However, unlike anxious women with BC, it is likely that non-anxious BC women felt able to effectively cope with appraised threat, hence effortfully directing action to actively manage the threat itself rather than the emotion evoked by it (Folkman, 2013). The use of adaptive engagement coping as reflected in conscious attentional vigilance may facilitate disconfirmatory information processing and so modify (or reflect less) pre-existing dysfunctional schemata, resulting in psychological adaption (Wells, 2013).

**TABLE 3:** Mean supraliminal attentional bias scores of the dot-probe task.

	Overall sample (*n* = 290)	Anxious BC (*n* = 31)	Anxious HC (*n* = 17)	Non-anxious BC (*n* = 109)	Non-anxious HC (*n* = 133)
	
	*M* (*SD*)	*M* (*SD*)	*M* (*SD*)	*M* (*SD*)	*M* (*SD*)
Supraliminal attentional bias using negatively valenced stimuli					
Stimulus (+ or −)/prime (BC or neutral)					
+/BC	0.42 (63.28)	−0.35 (57.48)	24.91 (84.37)	8.70 (45.19)	−9.30 (72.47)
+/Neutral	−1.87 (57.36)	7.19 (47.39)	−9.44 (81.66)	−1.28 (55.64)	−3.50 (57.63)
−/BC	8.20 (68.80)	7.15 (69.38)	28.55 (56.49)	2.96 (52.72)	10.14 (80.80)
−/Neutral*	4.55 (69.92)	−22.98 (49.03)	42.16 (105.20)	13.37 (59.48)	−1.07 (73.93)
Supraliminal attentional bias using cancer-related information					
Stimulus (Ca-related words or non−ca words)					
Ca-related words*	2.30 (43.63)	−17.84 (52.81)	−18.75 (29.89)	12.23 (43.39)	1.54 (40.62)
Non−ca words	−0.30 (48.28)	7.34 (56.58)	15.34 (68.89)	2.71 (31.66)	−6.60 (53.78)

+ positively valenced stimuli; − negatively valenced stimuli. BC, breast cancer; Ca-related words, cancer-related words; Non-ca words, non-cancer words. *p < 0.05.

Our findings also suggested that as expected, women with BC experiencing high or low anxiety relative to their healthy control counterparts had a greater tendency to overinterpret ambiguous information negatively. As discussed earlier, cancer experience may elaborate pre-existing cancer schemata among cancer survivors, which in turn enhanced the retrieval of CRI during interpretation processing (Beck and Clark, 1997; Clark and Beck, 1999). Such predisposition to negative, cancer-related interpretation in cancer survivors may further reinforce the activated dysfunctional schema by negative thoughts and images, causing and perpetuating anxiety (Beck, 1979). This also provided another possible explanation for the significant difference in anxiety between cancer survivors and healthy controls. However, interpretation bias did not differ between groups with high depression, probably due to the fact that negative interpretation bias was a common feature of depressed individuals (Everaert et al., 2017).

### Study Limitations

Several methodological limitations of this study merit comment. First, the BC sample was formed as a secondary analysis of our previous study (Lam et al., 2018). Second, with an average onset duration 4.75 years since diagnosis in the BC sample, distress levels may have varied over time. However, changes in attentional biases over time were less likely due to their stable, trait-like characteristics (Beck and Clark, 1997). Third, due to a single timepoint distress assessment in the healthy control sample, data were unavailable on duration of anxiety in the control group. We were only able to make a cross-sectional comparison of distress levels with the BC sample. Fourth, the small numbers of participants in high anxiety groups potentially weaken statistical power to detect smaller between-group differences (Lam et al., 2018). Fifth, the dot-probe task has been criticized as unreliable (Schmukle, 2005; Dear et al., 2011; McNally, 2018), yet the issue of reliability might be canceled out in studies comparing different samples (Schmukle, 2005). More importantly, this indirect reaction time-based method is insensitive for capturing visual temporal attention patterns (Möbius et al., 2018). The use of more direct and precise AB measurements, such as eye-tracking, should be used in future studies. Nevertheless, given its previously wide use as an AB assessment research, the use of the dot-probe task in this study enables cross-studies comparison within the narrower psycho-oncology literature (Butow et al., 2015). Sixth, the word stimuli used in the dot-probe task were not matched for the Chinese character frequency. Lastly, the reported findings are not generalizable to male or other cancer populations.

### Clinical Implication

As an extension of the original study, a generalization of conscious attentional avoidance response to general threat stimuli was identified as a distinctive feature of anxious women with BC. Hypersensitivity to potential threat in ambiguous cues due to elaborated dysfunctional schema potentially predisposed this subset of affected women to utilize avoidance coping strategies, which can impair emotional regulation in a long-term. Future studies and interventions could provide AB modification through promoting active goal-oriented attention search (Mogg and Bradley, 2016; Lam et al., 2018) to focus individuals’ attentional resources on the threat itself for threat habituation, or challenge pre-existing dysfunctional schema through information disconfirmatory processes to reduce threat salience, or modify threat appraisal through enhancing cancer patients’ coping self-efficacy, all of which perhaps promote the use of active coping approach for better distress management. For example, provision of self-management support (SMS), which has been recently listed as a priority area for action in cancer care, including interventions to boost patients’ self-efficacy and improve coping capacity, perhaps enables them in effective self-management and optimizes health outcomes (Howell et al., 2020). However, in practice, it remains challenging to ensure healthcare professionals actually offer such support. Along with already heavy workloads, a focus on biomedical aspects of disease and low confidence in discussing psychosocial aspects, little of which is included in training programs and even less in practice, with rare exceptions, means psychosocial support is often absent or minimal. A care model partnering with non-governmental organizations to provide staff coaching, and skills-based SMS workshops to cancer patient, with professional organizations emphasizing benefits from psychosocial interventions could help to facilitate implementation in clinical populations.

## Data Availability Statement

The raw data supporting the conclusions of this article will be made available by the authors, without undue reservation, to any qualified researcher.

## Ethics Statement

The studies involving human participants were reviewed and approved by the Institutional Review Board of the University of Hong Kong/Hospital Authority Hong Kong West Cluster (Ref: UW14-136). The patients/participants provided their written informed consent to participate in this study.

## Author Contributions

DN completed the data collection, performed the statistical data analysis, and drafted the initial version of the manuscript. WL and RF oversaw this research study, contributed to manuscript revision, and approved the final submitted version. All authors contributed to the article and approved the submitted version.

## Conflict of Interest

The authors declare that the research was conducted in the absence of any commercial or financial relationships that could be construed as a potential conflict of interest.
