# Supplementary material for: The Generalization of Conscious Attentional Avoidance in Response to Threat Among Breast Cancer Women With Persistent Distress
Source: Front Psychol. 2020 Dec 21;11:589088. doi: 10.3389/fpsyg.2020.589088 (PMC7779411; doi:10.3389/fpsyg.2020.589088)
Supplement: Supplementary file 1 [file Table_1.DOCX]

Supplementary materials

Word pairs used in the dot-probe task using negatively-valenced words

|  | Positive-Neutral word pairs | | Negative-Neutral word pairs | |
| --- | --- | --- | --- | --- |
|  | Positively-valenced | Neutral | Negatively-valenced | Neutral |
| 1 | 優點 (Strength) | 地面 (Ground) | 自殺 (Suicide) | 處境 (Situation) |
| 2 | 滿足 (Satisfied) | 普通 (Ordinary) | 虐待 (Abused) | 習俗 (Custom) |
| 3 | 讚美 (Praise) | 試驗 (Test) | 難受 (Uncomfortable) | 哪些 (Which) |
| 4 | 有益 (Useful) | 就是 (That is) | 難過 (Sadness) | 選擇 (Selection) |
| 5 | 出色 (Outstanding) | 普遍 (General) | 憂愁 (Sadness) | 訊息 (Message) |
| 6 | 喜愛 (Favourite) | 果然 (Really) | 痛哭 (Crying) | 器具 (Appliance) |
| 7 | 欣賞 (Appreciation) | 作風 (Style) | 孤立 (Isolated) | 化石 (Fossil) |
| 8 | 快活 (Happiness) | 例如 (For example) | 苦楚 (Suffering) | 等級 (Grade) |
| 9 | 漂亮 (Prettiness) | 徹底 (Thorough) | 疾病 (Disease) | 通知 (Notice) |
| 10 | 欣喜 (Happiness) | 估計 (Estimation) | 絕望 (Despair) | 題材 (Theme) |
| 11 | 歡笑 (Laughter) | 分配 (Distribution) | 淒涼 (Desolate) | 谷子 (Millet) |
| 12 | 喜悅 (Joy) | 一帶 (Area) | 委屈 (Wronged) | 固然 (of course) |
| 13 | 愉快 (Happiness) | 產量 (Yield) | 困苦 (Hardship) | 處於 (In) |
| 14 | 希望 (Hope) | 面前 (Before) | 分離 (Separation) | 軌道 (Track) |
| 15 | 優良 (Excellent) | 溫度 (Temperature) | 苦惱 (Distressed) | 地下 (Underground) |
| 16 | 鼓勵 (Encouragement) | 大約 (Approximately) | 心疼 (Distressed) | 筷子 (Chopsticks) |
| 17 | 歡喜 (Joy) | 情形 (Situation) | 喪氣 (Frustrated) | 人家 (People) |
| 18 | 傑出 (Outstanding) | 夜裡 (At night) | 折磨 (Torture) | 大眾 (Public) |
| 19 | 喜歡 (Like) | 會議 (Meeting) | 艱苦 (Hard) | 總結 (Summary) |
| 20 | 美麗 (Beauty) | 環境 (Surroundings) | 悲慘 (Tragic) | 饅頭 (Steamed bread) |
| 21 | 慶祝 (Celebration) | 程度 (Degree) | 困境 (Dilemma) | 東西 (Thing) |
| 22 | 優秀 (Excellent) | 方針 (Policy) | 打仗 (Fight) | 事件 (Event) |
| 23 | 讚揚 (Praise) | 設計 (Design) | 憤怒 (Anger) | 形象 (Image) |
| 24 | 幸福 (Happiness) | 職級 (Rank) | 錯誤 (Error) | 裡面 (Inside) |
| 25 | 親愛 (Dear) | 原因 (Reason) | 苦悶 (Depressed) | 寫作 (Writing) |
| 26 | 開心 (Happiness) | 椅子 (Chair) | 悲觀 (Pessimistic) | 建造 (Construct) |
| 27 | 稱讚 (Compliment) | 專門 (Specialized) | 受罪 (Suffer) | 報名 (Sign up) |
| 28 | 喜訊 (Good news) | 方案 (Proposal) | 煩惱 (Upset) | 鄰居 (Neighbour) |
| 29 | 優越 (Superior) | 標準 (Standard) | 創傷 (Trauma) | 印染 (Printing) |
| 30 | 感激 (Gratefulness) | 從此 (Since then) | 戰爭 (War) | 民族 (Nation) |
| 31 | 快樂 (Happiness) | 附近 (Nearby) | 衰弱 (Weak) | 營業 (Business) |
| 32 | 甜蜜 (Sweetness) | 一半 (Half) | 疼痛 (Pain) | 調動 (Move) |

Word pairs used in the dot-probe task using cancer-related information

|  | Cancer-Neutral word pairs | | Positive-Neutral word pairs | |
| --- | --- | --- | --- | --- |
|  | Cancer-related | Neutral | Positive | Neutral |
| 1 | 乳癌 (Breast cancer) | 氣溫 (Temperature) | 優點 (Strength) | 營房 (Barracks) |
| 2 | 乳房 (Breast) | 事迹 (Achievement) | 滿足 (Satisfied) | 特地 (Specially) |
| 3 | 癌症 (Cancer) | 探測 (Detection) | 讚美 (Praise) | 譬如 (For example) |
| 4 | 水腫 (Edema) | 田地 (Field) | 有益 (Useful) | 蚯蚓 (Earthworm) |
| 5 | 淋巴 (Lymph) | 樞紐 (Hub) | 出色 (Outstanding) | 措施 (Measures) |
| 6 | 疤痕 (Scar) | 當兒 (At the time) | 喜愛 (Favourite) | 因素 (Factor) |
| 7 | 手術 (Surgery) | 即將 (Soon) | 欣賞 (Appreciation) | 可見 (Visible) |
| 8 | 腫瘤 (Tumor) | 包含 (Inclusion) | 快活 (Happiness) | 功夫 (Effort) |
| 9 | 硬塊 (Lumps) | 背景 (Background) | 漂亮 (Prettiness) | 典型 (Typical) |
| 10 | 切除 (Resection) | 彈簧 (Spring) | 欣喜 (Happiness) | 平方 (Square) |
| 11 | 重建 (Reconstruction) | 協會 (Association) | 歡笑 (Laughter) | 出席 (Attend) |
| 12 | 矯形 (Orthopaedic) | 書本 (Book) | 喜悅 (Joy) | 錄音 (Recording) |
| 13 | 義乳 (Breast prosthesis) | 值班 (On duty) | 愉快 (Happiness) | 手勢 (Gesture) |
| 14 | 電療 (Radiation therapy) | 事物 (Thing) | 希望 (Hope) | 下巴 (Chin) |
| 15 | 化療 (Chemotherapy) | 少數 (Minority) | 優良 (Excellent) | 最終 (Finally) |
| 16 | 標靶 (Target therapy) | 必然 (Certainty) | 鼓勵 (Encouragement) | 依照 (According to) |
| 17 | 治療 (Treatment) | 加入 (Join) | 歡喜 (Joy) | 半夜 (Midnight) |
| 18 | 疲倦 (Tiredness) | 夜間 (Night-time) | 傑出 (Outstanding) | 形勢 (Situation) |
| 19 | 噁心 (Nausea) | 手工 (Manual) | 喜歡 (Like) | 包括 (Inclusion) |
| 20 | 反胃 (Nausea) | 行列 (Rank) | 美麗 (Beauty) | 電流 (Current) |
| 21 | 嘔吐 (Vomiting) | 積雪 (Snow) | 慶祝 (Celebration) | 混紡 (Blended) |
| 22 | 脫髮 (Hair loss) | 顆粒 (Particles) | 優秀 (Excellent) | 起重 (Lifting) |
| 23 | 頭昏 (Dizziness) | 辨別 (Distinguish) | 讚揚 (Praise) | 茶館 (Teahouse) |
| 24 | 痛楚 (Pain) | 設置 (Set up) | 幸福 (Happiness) | 鋼板 (Steel plate) |
| 25 | 發熱 (Fever) | 前列 (forefront) | 親愛 (Dear) | 聯絡 (Connection) |
| 26 | 肩痛 (Shoulder pain) | 面貌 (Face) | 開心 (Happiness) | 絲毫 (Slightest) |
| 27 | 麻痺 (Paralysis) | 古代 (Ancient) | 稱讚 (Compliment) | 路程 (Distance) |
| 28 | 傷口 (Wound) | 聯合 (Joint) | 喜訊 (Good news) | 行使 (Exercise) |
| 29 | 藥物 (Medicine) | 在內 (Inside) | 優越 (Superior) | 結論 (Conclusion) |
| 30 | 骨針 (Bisphosphonates) | 比方 (Example) | 感激 (Gratefulness) | 市場 (Market) |
| 31 | 復發 (Recurrence) | 紙張 (Paper) | 快樂 (Happiness) | 大批 (A large number of ) |
| 32 | 擴散 (Metastasis) | 集會 (Assembly) | 甜蜜 (Sweetness) | 水銀 (Mercury) |

A list of word stimuli used in the ambiguous cue task

|  | Unambiguous words | Ambiguous words |
| --- | --- | --- |
| 1 | 天 (Sky) | 腫 (Swollen) |
| 2 | 理 (Reason) | 脹 (Swell) |
| 3 | 草 (Grass) | 癌 (Cancer) |
| 4 | 工 (Work) | 毒 (Poison) |
| 5 | 家 (Family) | 乳 (Breast) |
| 6 | 日 (Day) | 傷 (Hurt) |
| 7 | 火 (Fire) | 胸 (Breast) |
| 8 | 月 (Moon) | 瘤 (Tumor) |
| 9 | 大 (Big) | 療 (Treatment) |
| 10 | 花 (Flower) | 化 (Chemotherapy) |
| 11 | 車 (Car) | 疤 (Scar) |
| 12 | 晴 (Sunny) | 痛 (Pain) |
| 13 | 美 (Nice) | 淋 (Lymph) |
| 14 | 風 (Wind) | 醫 (Medical) |
| 15 | 舒 (Leisure) | 藥 (Medicine) |
| 16 | 境 (Boundary) | 亡 (Death) |
| 17 | 人 (People) | 復 (Recurrence) |
| 18 | 開 (Open) | 發/髮 (Hair) |
| 19 | 因 (Because) | 擴 (Metastasis) |
| 20 | 分 (Minute) | 散 (Metastasis) |
| 21 | 路 (Road) | 手 (Hands) |
| 22 | 安 (Safety) | 痺/臂 (Paralysis/ Arm) |
| 23 | 然 (Then) | 暈 (Dizziness) |
| 24 | 事 (Thing) | 吐 (Vomiting) |
| 25 | 色 (Colour) | 電 (Radiation therapy) |
